# Epigenetic Regulation of Elf5 Is Associated with Epithelial-Mesenchymal Transition in Urothelial Cancer

**DOI:** 10.1371/journal.pone.0117510

**Published:** 2015-01-28

**Authors:** Bo Wu, Xiaoming Cao, Xuezhi Liang, Xuhui Zhang, Wei Zhang, Guang Sun, Dongwen Wang

**Affiliations:** 1 Department of Urology, First Hospital of Shanxi Medical University, Taiyuan, China; 2 First Clinical Medical College, Shanxi Medical University, Taiyuan, China; 3 Department of Urology, Second Hospital of Tianjin Medical University, Tianjin, China; University of Nebraska Medical Center, UNITED STATES

## Abstract

E74-like factor 5 (Elf5) has been associated with tumor suppression in breast cancer. However, its role in urothelial cancer (UC) is completely unknown. Immunohistochemistry (IHC) and methylation specific PCR (MSP) were done to detect Elf5 expression level and its promoter methylation. Results revealed that low expression of Elf5 on protein and mRNA levels were associated with tumor progression, early relapse and poor survival. In vitro, down-regulation of Elf5 can increase epithelial-mesenchymal transition (EMT). Aberrant Elf5 methylation was identified as major mechanism for Elf5 gene silence. Accordingly, restoration of Elf5 by infection or demethylating treatment effectively reversed EMT processes. In conclusion, we identified Elf5 as a novel biomarker of UC on several biological levels and established a causative link between Elf5 and EMT in UC.

## Introduction

The EMT affects critical steps of marked changes in cell adhesion, polarity and migratory by interconverting epithelial cells into cells with mesenchymal / stem cell (SC) state [1,2,3]. EMT and its reverse program, mesenchymal-epithelial transition (MET), were activated in tumour cells and this progress enable these cells to acquire cellular traits associated with high-grade, invasiveness and recurrence [3,4,5]. Given the complexity and dynamic nature of EMT, it is impossible to be maintained by a single signaling pathway. TGF-β, Wnt, Notch, Sonic Hedgehog, EGF and FGF pathways have been proved to be important for governing these transitions in cancer development and progression [2,3,6,7]. More recently, epigenetic modifications such as methylation / demethylation have also been shown to be involved in EMT [8,9,10]. However, the signaling mechanisms that induce and maintain this mesenchymal / SC state still remain unclear.

Elf5 is a member of the epithelium specific subgroup of the large E-twenty-six (ETS) transcription factor family. It has been proved as a key transcriptional determinant of breast cancer lineage by suppressing estrogen sensitivity in luminal cells and enhancing basal characteristics in basal breast cancer cells [11]. More recent studies show Elf5 is not only as a key cell lineage regulator, but also as a suppressor of EMT by repressing the transcription of Snail2 [12]. In addition, Elf5 gene silencing is triggered by dynamic promoter hypermethylation in the processes of human placenta development and embryonic cell lineage formation [13,14]. However, the roles of Elf5 in urothelial cancer (UC) development and progression remain undefined.

Bladder UC is the sixth most common cancer in the world with about 386,000 new cases in 2008 and estimated 150,000 deaths [15]. At the first diagnosis of UC, nearly 80% patients present with non-muscle invasive bladder cancer (NMIBC) while the remainders with muscle invasive diseases [16,17]. Of these patients with NMIBC, 50%–70% will have at least one recurrence in 5 years, and more than 20% will exhibit muscle infiltration [18] and cause a mortality rate up to 50% within 2 years of diagnosis [19]. The issue associated with UC is their highly unpredictable potential for recurrence and progression. Therefore, we wished to determine whether epigenetic inactivation of Elf5 is associated with UC progression and early recurrence.

## Materials and Methods

### Ethics statement, Patients and Samples

Cohort 1:

A total of 182 formalin-fixed paraffin-embedded bladder UC samples were obtained among the 275 consecutive patients between 2004 and 2008 from the pathology departments of Tianjin Institute of Urology (Table 1). 10 contemporary formalin-fixed paraffin-embedded normal urothelium (NU) were also obtained. Among the analyzed patients, 115 primary bladders UCs underwent transurethral resection of bladder tumor (TURBT) (***Cohort 1A***) and the remained 67 patients underwent radical cystectomy (***Cohort 1B***). None of the primary patients received any preoperative anticancer treatment. Follow-up information was obtained from the medical records of patients who fulfilled study inclusion criteria. In brief, patients presented postoperatively every 3 months for the first 2 years after therapy and semiannually thereafter. Recurrence was defined as a new tumor observed in the bladder after TURBT. Recurrence-free survival (RFS) was calculated as the time from TURBT to the date of recurrence. Disease-specific survival (DSS) was calculated as the time from radical cystectomy to the date of death from UC. Mean follow-up was 49.4 months (range 6 to 90) and 56.4 months (range 6 to 106) in ***Cohort 1A*** and ***Cohort 1B***, respectively.

**Table 1:** Clinical characteristics and ELF5 expression in *cohort 1* patients.

Variable		No. of		ELF5			P
		patients		≤10%	>10%		
Normal tissue		10		1	9		0.01
TCC		182		103	79		
Age, years							
<60		77		45	32		0.67
≥60		105		58	47		
Tumor size, cm							
<3		113		61	52		0.36
≥3		69		42	27		
Tumor grade							
G_1–2_		67		31	36		0.03
G_3_		115		72	43		
Tumor stage							
T_a_-T_1_		146		77	69		0.03
T_2_-T_4_		36		26	10		
Tumor occurrence							
Primary		139		73	66		0.04
Recurrent		43		30	13		

Cohort 2:

A series of 10 NU and 50 UC tissues were obtained from retrospective record of Tianjin Institute of Urology. These tissues were flash-frozen in liquid nitrogen and stored at -80°C between 2010 and 2013. There was no confirmed recurrence and death in the study.

The pathologic diagnoses and the clinic-pathologic factors of the two cohorts were established based on the general guideline for primary bladder cancer as defined by the 2002 TNM classification and 1973 WHO grading system by experienced pathologists. The study protocol was approved by the ethics committee of Second Hospital of Tianjin Medical University, and written informed consent for the use of the specimens from each patient enrolled was obtained accordingly. All tissue samples and medical record data were anonymized prior to access and analysis by the researchers.

### Cell culture and treatment

The human bladder cell lines UROtsa, RT112, HT1376, J82 and T24 were originally obtained from the ATCC. The cell line EJ was a gift from Dr. Ruifa Han (Tianjin Medical University, China. He obtained the cell line from the ATCC). UROtsa was cultured in DMEM (Gibco, Shanghai). HT1376 was cultured in Eagle’s Minimum Essential Medium (EMEM, ATCC). Other cell lines were maintained in RPMI 1640 (Gibco, Shanghai). All culture mediums were supplemented with 10% Fetal Bovine Serum (FBS, Gibco, Melbourne) and 1% penicillin/streptomycin. Mycoplasma infection was regularly tested using the PlasmoTest^TM^ (InvivoGen, San Diego, CA). When necessary, cells were treated with 5μM 5-Aza-2′-deoxycytidine (5-AZA, Invivogen) according to previously determined concentration for 6 days of incubation [20,21].

### RNA extraction and Quantitative RT-PCR

RNA was isolated using the GenElute (Sigma-Aldrich, Shanghai) according to the manufacturer’s instructions. Reversed transcription was performed using Transcriptor First Strand cDNA Synthesis Kit (Roche, Shanghai). RT-PCR was performed on a BioRad iCycler iQ (Bio-Rad Laboratories) PCR machine (Applied Biosystem) using SYBR Green Master Mix. The gene-specific primer sets were used at a final concentration of 0.2μM and their sequences are listed in S1 Table. Each sample was run and analyzed in triplicate.

### Bisulfite-modification and MSP

Genomic DNA was obtained from tissue specimens using DNeasy Tissue Kit (Qiagen, Valencia, CA) following the manufacturer’s protocol. The extracted DNA was treated with bisulfite to convert unmethylated cytosines to uracils prior to methylation-specific polymerase chain reaction (MSP) using EpiTect Bisulfite Kit (Qiagen) according to the manufacture’s instruction. Modified DNA was amplified using MSP primers (S1 Table), which specifically recognized either the unmethylated (product size 258 bp) or methylated (258 bp) Elf5 promoter sequence after bisulphite conversion. Normal DNA from human appendix vermiformis was treated in vitro with SssI methyltransferase (New England Biolabs, Beverly, MA) to generate a positive control for methylated alleles [22].

### IHC staining

Paraffin-embedded sections (4μm) were deparaffinized and hydrated in xylene followed by graded alcohols to water. Antigen retrieval was performed in 0.01 M citrate for twice 10 min in a microwave oven followed by a 60-min cool down. Slides were then incubated with various primary antibodies followed by Envision-plus labeled polymer-conjugated horseradish peroxidase and DAB monitoring staining (Zhong Shan gold bridge, Beijing). Then, slides were counterstained and dehydrated for viewing and imaging. Antibodies were: anti-Elf5 (Santa Cruz Biotechnology, Santa Cruz, CA), anti-E-cadherin (Abcam, Cambridge, MA), anti-N-cadherin (Abcam), anti-Vimentin (Abcam).

### Scoring of IHC staining

Elf5 score: The entire section was evaluated by two respective observers being unaware of the clinical data. The tumor cells were determined positive when a clearly visible staining was detected in nucleus. NU tissues were included to be as internal controls. Percent of staining positive cell was graded as 1% to 100% with a 5% interval. Counted percents were rounded to the nearest gears. A cut-off value of 10% was determined arbitrarily, and according to this value, two groups (low and high Elf5 staining) were assigned.

Vimentin and E-cadherin score: Five random low magnification fields were selected, then the proportion of positive region was determined by ImageJ program. Cut-off values were determined by the median, according which, two groups (low and high expression) were assigned.

### Molecular cloning and viral infection

The pLEX plasmids (Open Biosystems) containing complementary DNAs for control GFP and Elf5 were generated by routine molecular cloning techniques. The HA-tagged WT Elf5 cDNAs was subcloned from pCMV-HA vector to pLEX plasmids using BamH1-Not1 restriction enzymes. Lentivirus production was performed essentially as previously described [23]. Virally infected cells were selected with puromycin.

### siRNA knockdown

For knockdown experiments, siRNA targeting the Elf5 gene (5’-AGCCCTGAGATACTACTATAA-3’, catalogue number GS2001) and siRNA negative control were purchased from Qiagen. Then, transfections were conducted using Lipofectamine RNAiMAX (Invitrogen).

### Immunoblotting

Protein was extracted with RIPA lysis buffer. Protein lysates were resolved on 10% Bis-Tris Gel, transferred to PVDF membranes, probed with HRP-linked secondary antibodies (GE Healthcare), and visualized with ECL reagent (Thermo Scientific). Antibodies were: anti-Elf5 (Santa Cruz Biotechnology, Santa Cruz, CA), anti-E-cadherin (Abcam, Cambridge, MA), anti-N-cadherin (Abcam), anti-Vimentin (Abcam), anti-Snail (Cell Signaling Technology, Beverly, MA), anti-ZEB1 (Abcam).

### Cell viability assay

Indicated cells were seeded in 24-well plates at a density of 5,000 per well in medium containing 10% FBS. At the indicated time point, medium was removed and serum-free medium containing 3-(4,5-dimethylthiazol-2-yl)-2,5-diphenyltetrazolium bromide (MTT; 0.5 mg/mL; Sigma) was then added into each well. Four hours after incubation at 37°C, cellular formazan products were dissolved with acidic isopropanol, and the absorbance at 570 nm was measured by spectrophotometry (Beckman Du640B). Six replicate wells were used for each sample at each time point.

### Migration assays

Twenty-five thousand cells were seeded into 24-well cell culture inserts with 8 mm pores (BD Falcon). After 24–48 hr, the cells on the upper surface of the filters were removed with a cotton swab. For visualization, cells on lower filter surfaces were fixed and stained with a toluidine blue. Entire fields of filter were counted. Data are presented as migrated cells per field.

### Sphere assay

Assays were performed as previously described with modifications [24]: 1000 cells/well were seeded in 6-well ultra-low adhesion plates (Costar) in MEGM medium containing 10% Serum Replacement (KnockOut) supplemented with 1× MEM non-essential amino acid (Gibco), 20 ng/ml EGF (R&D Systems), 10 ng/ml bFGF (R&D Systems), and B27 (Gibco).

### Statistical analysis

Data were reported as mean ± SE unless otherwise stated. The one-way ANOVA and Student *t* test were used to analyze the differences among/between groups, respectively. SPSS (v.19, IBM, USA) was used to assess date. *P*<0.05 was considered statistically significant.

## Results

### Elf5 mRNA expression is decreased in human bladder urothelial cancer

**Fig 1:**
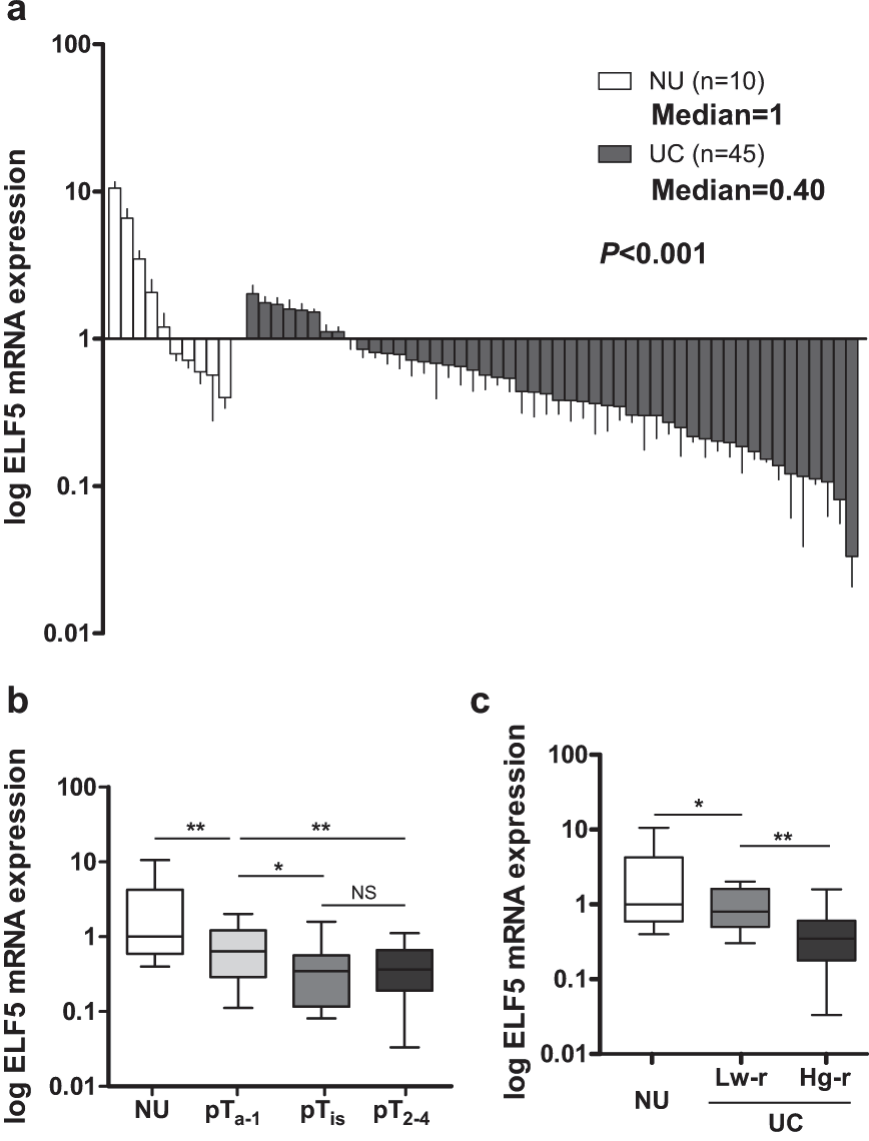
Low expression of Elf5 mRNA in human bladder UC. **a**, Elf5 mRNA expression is investigated in 50 UC and 10 NU tissue samples by Real-time PCR. Data are presented as mean ± standard error of the mean (SEM). **b and c**, Box plots demonstrate significantly down-regulated Elf5 mRNA in both invasive (pT2–4) and high-risk UCs (Hg-r). Horizontal lines: grouped medians. Boxes: 25–75% quartiles. Vertical lines: minimum and maximum; NS: not significant, *P < 0.05, **P < 0.01. Low-risk UC (Lw-r): low grade pTa; Hg-r UC: High grade pTa, CIS and invasive UC. The results shown are from two or three independent experiments.

Relative Elf5 levels in tumor tissue from 50 UC patients were determined by qRT-PCR spanning 90 bp, normalized by GAPDH, and were compared with the median of 10 normal tissue control samples (Fig. 1a). Relative Elf5 expression in normal tissues was variable within a range of 0.39 to 10.56-fold of median levels. Tumor samples showed a decrease in Elf5 levels within a range of 0.03 to 2.02-fold in comparison to the same control as normal tissues, with a median of 0.40-fold lower expression (Fig. 1a). To determine whether Elf5 level showed any significant difference among different tumor stages, the fold change in Elf5 expression was calculated. Intermediate reduction of Elf5 mRNA was detected in NMIBC and low risk UC, while highly significant loss of Elf5 mRNA expressions was observed in invasive (pT2–4, *P*<0.01, Fig. 1b) and high risk UC (High grade pTa, CIS and invasive UC, *P*<0.01, Fig. 1c), compared with NMIBC and low risk UC (low grade pTa), respectively. Based on these results, decreased Elf5 expression appears to be an important event in UC development and progression.

### Elf5 protein is decreased in urothelial Cancer and associated with tumor progression

**Fig 2:**
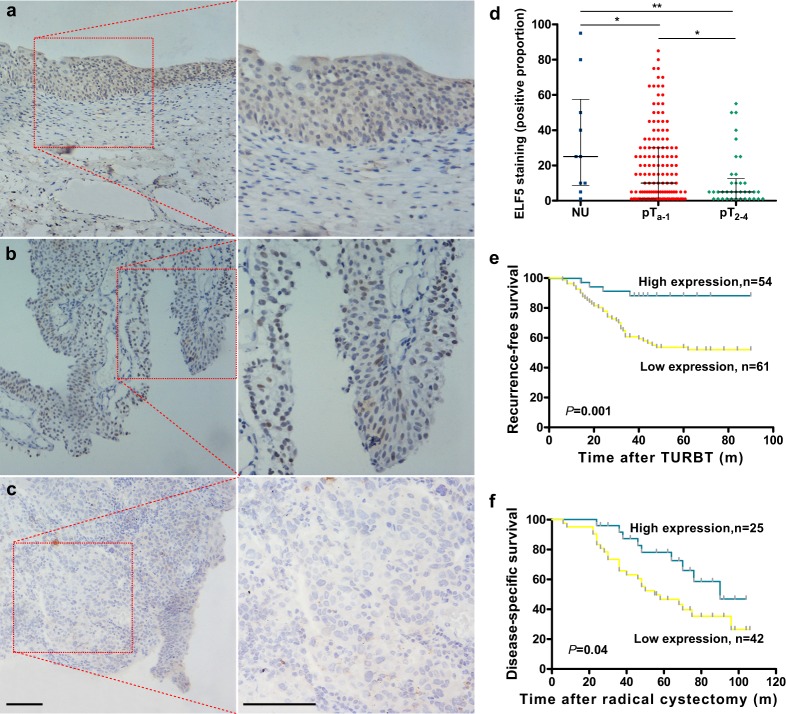
Elf5 protein is down-regulated expression and prognostic for RFS and DSS in UC. **a**, Representative strong Elf5 IHC staining in epithelial cells of NU (85%). Scale bars, 100mm. **b**, Representative moderate Elf5 staining in non-invasive pTa tumors (30%). **c**, Representative weak staining in invasive NU (<5%). **d**, Box plot shows Elf5 IHC staining in NU (n = 10), pTa-1 (n = 146) and invasive UCs (n = 36). Horizontal lines: grouped medians. Boxes: 25–75% quartiles. Vertical lines: minimum and maximum; *P < 0.05, **P < 0.01. **e**, Kaplan-Meier curves of recurrence-free survival (RFS) according to Elf5 staining level in primary NMIBC treated by TURBT. **f**, Kaplan-Meier curves of disease-specific survival (DSS) according to Elf5 staining level in UC patients after radical cystectomy.

Next, immunohistochemistry analyses of human NU and UC specimens were performed to verify whether Elf5 expression decreased in UC on protein level. IHC staining was quantified by counting proportion of positive-stained nucleus. In line with the mRNA data, we found Elf5 protein was stained in most normal urothelial epithelial cells (Fig. 2a). In contrast, Elf5 expression was prominently decreased in UC tissues (Fig. 2b) and almost completely lost in invasive tumors (Fig. 2c). Then, the Elf5 expression was quantified according to tumor stages. Strong Elf5 staining was demonstrated in NU samples with a mean of 34.10±10.21 positive rate, whereas moderate and weak Elf5 staining were observed in pTa (19.36±1.77, *P*<0.05) and invasive UCs (11.03±2.56, *P*<0.01, Fig. 2d), respectively. This suggested that Elf5 silencing may contribute to UC invasiveness. We further evaluated the prognostic value of Elf5 in two clinical databases (***Cohort 1A and Cohort 1B***) of UC by univariate Kaplan-Meier analysis. Cohort 1A was composed of patient with NMIBC, who received therapy of TURBT. Patients from this group are rarely confronted with death, but always disease relapse, so the RFS was used. We found that patients with high Elf5 expression tend to have better RFS (HR = 0.34, *P* = 0.001, Fig. 2e). In contrast, patients from cohort 1B tend to be confronted with death, so the DSS was selected. Result also revealed significant trend toward favorable prognosis for Elf5-high patients (HR = 0.49, *P* = 0.04, Fig. 2f). In addtion, results based on cut-offs of 5% and 15% were also conducted (S1 Fig.). The same trends with similar results were obtained, although a non-significantly higher DSS was detected in patients with Elf5 expression more than 15%. Overall, our clinical data support the supposed role that Elf5 may function to oppose bladder cancer progression.

### Elf5 knockdown in epithelial-like HT1376 cells induces EMT

**Fig 3:**
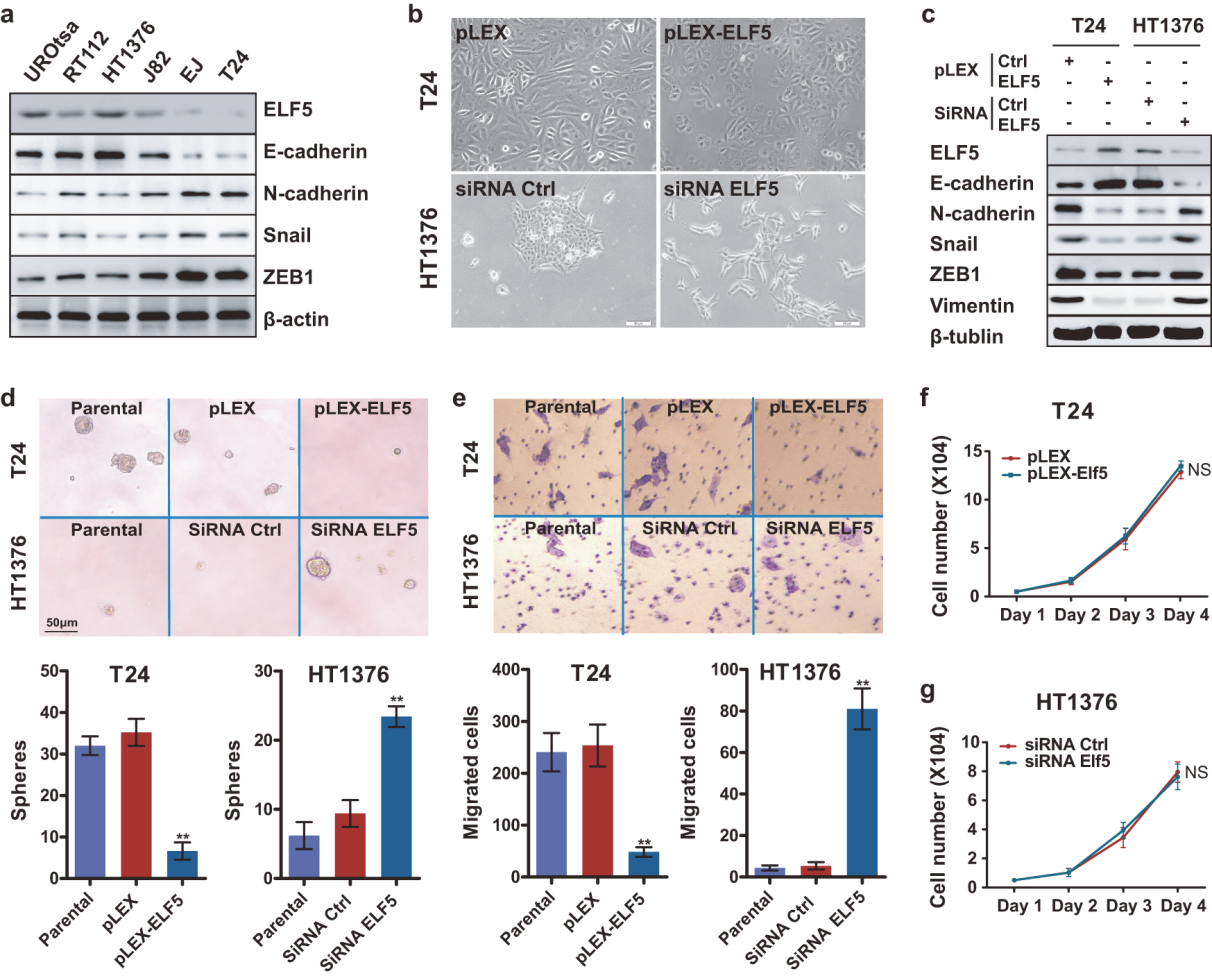
Negative correlation between Elf5 and EMT in UC cells. **a**, Elf5 and EMT markers are detected by Western blot analysis in urothelial cell lines. **b**, Bright-phase microscopy: T24 cells transduced with vector or HA-Elf5 and HT1376 cells treated with control or Elf5 siRNA. **c**, Western blot analyses of Elf5 and EMT markers in T24 cells transduced with vector or HA-Elf5 and HT1376 cells treated with control or Elf5 siRNA. **d and e**, Sphere assay (n = 6) and Migration assay (n = 3): bright-phase microscopy images and quantification of parental T24, T24-vector, T24-Elf5, and parental HT1376 as well as control and Elf5-siRNA treated HT1376 cells. Data are presented as mean±SEM, **P < 0.01.**f and g**, Cell viability was determined using MTT assay. Data are shown as mean±SEM. Six replicate wells were performed for each sample at each time point.

Previous evidence has revealed a mechanism of Elf5 inhibiting EMT in breast cancer [12]. To investigate if Elf5 can also regulates EMT in the context of UC, we tested Elf5 expression patterns in a panel of urothelial cell lines. Significantly low level of Elf5 was noticed in cell lines characterized by mesenchymal markers (EJ and T24), mesenchymal-like cells, suggesting a potential inhibitory role of Elf5 in EMT (Fig. 3a). In epithelial-like UROtsa, RT112 and HT1376 cells (identified by the high expression of epithelial markers), high level of Elf5 expression was demonstrated (Fig. 3a). To test the possible relationship between Elf5 and EMT, short interfering RNAs (siRNAs) were used to knock down Elf5 in epithelial-like HT1376 cells. In contrast to the epithelial island-forming, parental cells, the siRNA-treated cells consisted of front-to-back polarized, single cells (Fig. 3b). Similar to previously reported EMT cells [6,12], siRNA-treated cells expressed reduction in E-cadherin and increase in N-cadherin, vimentin as well as EMT-promoting transcription factors (eg. Snail and ZEB1) (Fig. 3c).

The sphere assay measures anchorage-independent proliferation at clonal density in vitro has been associated with the presence of epithelial-mesenchymal state [6]. We therefore examined sphere-forming abilities of HT1376 cells to gauge their status of EMT. Relative to the parental and control siRNA (siRNA Ctrl) cells, silencing of Elf5 were 3-fold enriched in sphere-forming cells (Fig. 3d). To test another functional hallmark of the mesenchymal state, we conducted in vitro invasive assays. Elf5-knockdown cells were more invasive (18-fold compared to parental and control cells, Fig. 3e). Furthermore, cell viability assays by MTT confirmed that the effect of Elf5 silence on sphere-forming ability and invasiveness is not a consequence of increased cell number (Fig. 3f and 3g). Together, our observations indicate that Elf5 is an inhibitor of EMT in UC cells.

### Elf5 promotes MET in mesenchymal-like T24 cells

Meanwhile, the relationship between Elf5 and EMT was confirmed by stable overexpression of Elf5 in mesenchymal-like T24 cells, which don’t express detectable endogenous Elf5 (Fig. 3a). We found Elf5 overexpression effectively increased formation of epithelial island in T24 cells (Fig. 3b). In addition, Elf5 overexpression decreased the expression level of several mesenchymal cell markers, such as N-cadherin and vimentin, and increased expression of E-cadherin (Fig. 3c). Furthermore, decreased EMT-related transcription factors were also detected in Elf5-overexpressed T24 cells (Fig. 3c). Functionally, 5-fold sphere-forming abilities and 4-fold invasiveness were decreased after stable enforced-expression of Elf5 in T24 cells compare with empty plasmid group (Fig. 3d and 3e). Elf5-induced MET was further confirmed in PC3 cells, a mesenchymal-like prostate cancer cell line with powerful invasiveness (data not shown). Collectively, our observations indicate that Elf5 is an enforcer of MET in UC cells.

### Elf5 is associated with E-cadherin and vimentin expression in human UC

**Fig 4:**
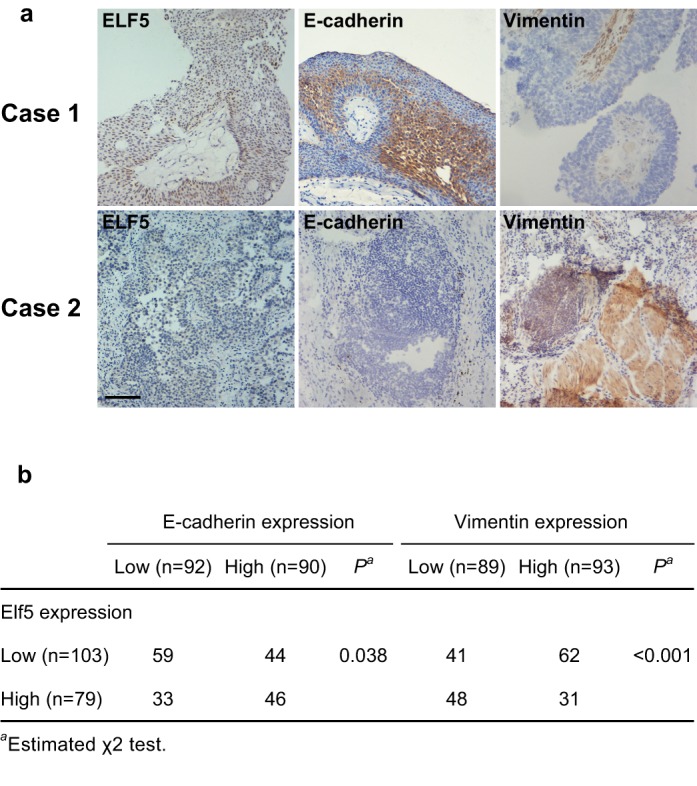
Correlation between Elf5 expression and E-cadherin/vimentin expression in 182 UC patients. A and b, IHC staining of E-cadherin and vimentin in representative cases with high (case 1) and low Elf5 expression sample (case 2). Scale bars, 100mm. b, Correlation analysis of Elf5 and EMT markers E-cadherin/vimentin.

To confirm the in vitro finding that the Elf5 is an inhibitor of EMT and an enforcer of MET in UC cells, we analyzed the expression levels of Elf5, E-cadherin and vimentin by IHC in ***Cohort 1*** patient samples (Fig. 4a). Low expression of Elf5 (e.g., case 2) is detected in 56.6% (103 of 182) of the samples and significantly associated with low E-cadherin expression (*P*<0.05) and high vimentin expression (*P*<0.05, Fig. 4b). These data suggest that the Elf5 is inversely associated with mesenchymal phenotype in patients with UC.

### Elf5 promoter hypermethylation inversely correlates with Elf5 mRNA expression in human UC

**Fig 5:**
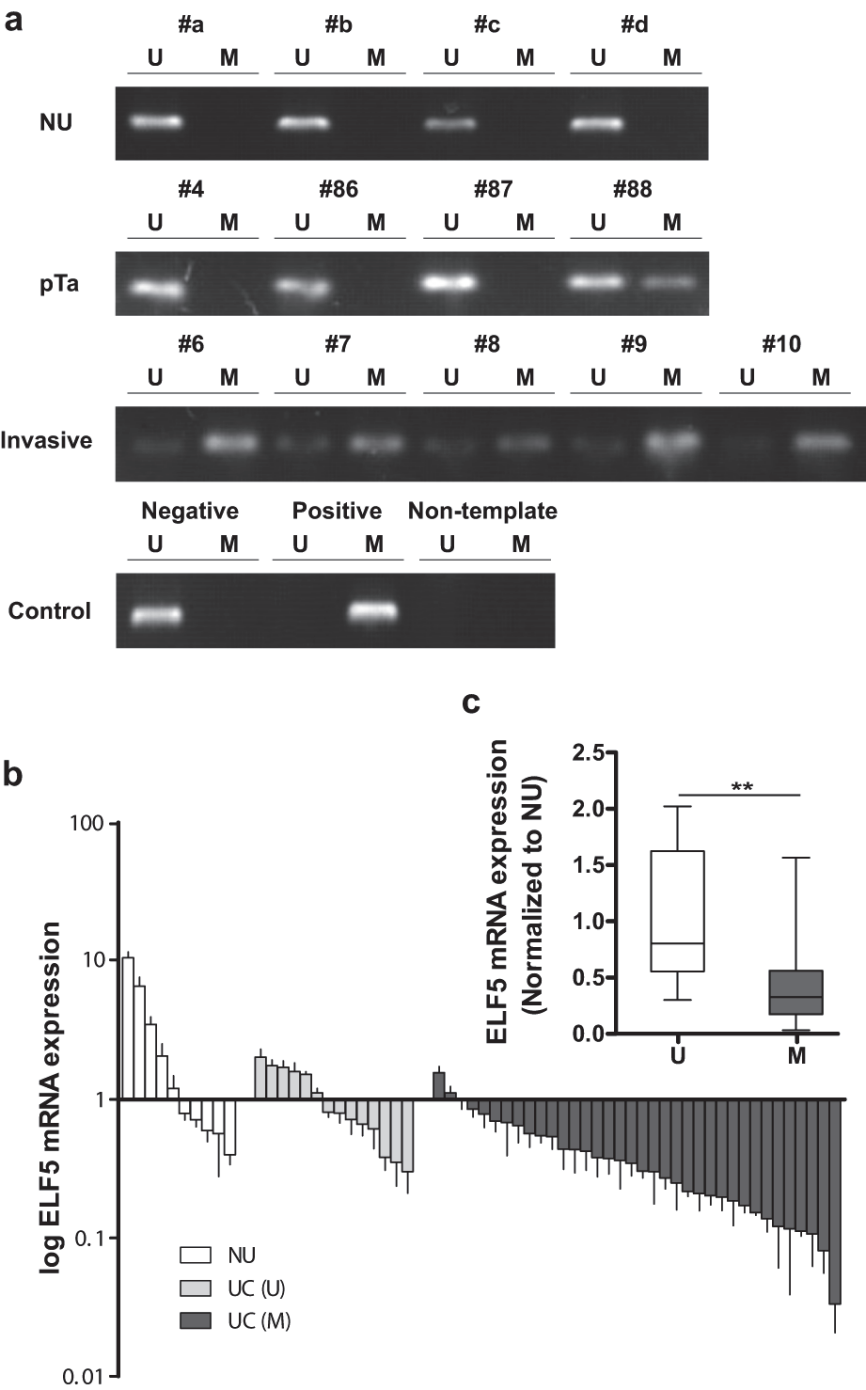
Negative correction between Elf5 promoter hypermethylation and mRNA expression in human bladder samples. a, Representative MSP results of the Elf5 promoter methylation in NU, pTa and invasive UC tissue samples as well as in negative and positive controls. U and M represent unmethylated and methylated DNA. Bisulphite-converted unmethylated genomic, polymethylated genomic DNA and non-template are used as controls. b, Elf5 mRNA expression by Real-time PCR is investigated according to Elf5 promoter methylation status in 10 NU and 50 UC tissue samples. Data are presented as mean ±SEM. c, Box plot shows loss of Elf5 mRNA expression in patients with methylated Elf5 promoter. Horizontal lines: grouped medians. Boxes: 25–75% quartiles. Vertical lines: minimum and maximum. **P < 0.01.

After the loss expression of Elf5 in human UC tissues is acknowledged, we further go to query the potential reasons. Recent study has demonstrated that placental Elf5 expression is down-regulated with gestational age increase, and epigenetic regulation was proved to be as ‘gatekeeper’ [13]. We then tested whether the activity state of Elf5 in human UC is also epigenetically controlled by its DNA methylation. Firstly, we designed primers for MSP using MethPrimer [25]. MSP for sequence of Elf5 promoter between -1000 bp and the transcription start site showed only one methylation in 10 NU samples (1/10, Fig. 5a). Subsequently, we analyzed methylation status of Elf5 promoter in bladder cancer samples (***Cohort 2***, n = 50) (Fig. 5a), and found a methylation frequency of 72% (36/50). To determine whether Elf5 promoter methylation contributes to Elf5 expression loss in UC, we correlated methylation and mRNA expression level in patients from ***Cohort 2***. As in Fig. 1a, 10 NU tissues served as control and their median expression rate was set to absolute value 1.0. Compared with these NU tissues, unmethylated UC showed an almost equal Elf5 expression without considering tumor stage or grade (median: 0.80, *P* = 0.08, Fig. 5b). In contrast, methylated tumor tissues showed a significant loss of Elf5 mRNA expression (median: 0.33, *P*<0.01, Fig. 5b). This correlation between loss of Elf5 mRNA expression and Elf5 promoter methylation suggests aberrant Elf5 methylation appears to be a major mechanism for Elf5 gene silencing in UC.

### Targeting promoter methylation leads to Elf5 expression in UC cell lines

**Fig 6:**
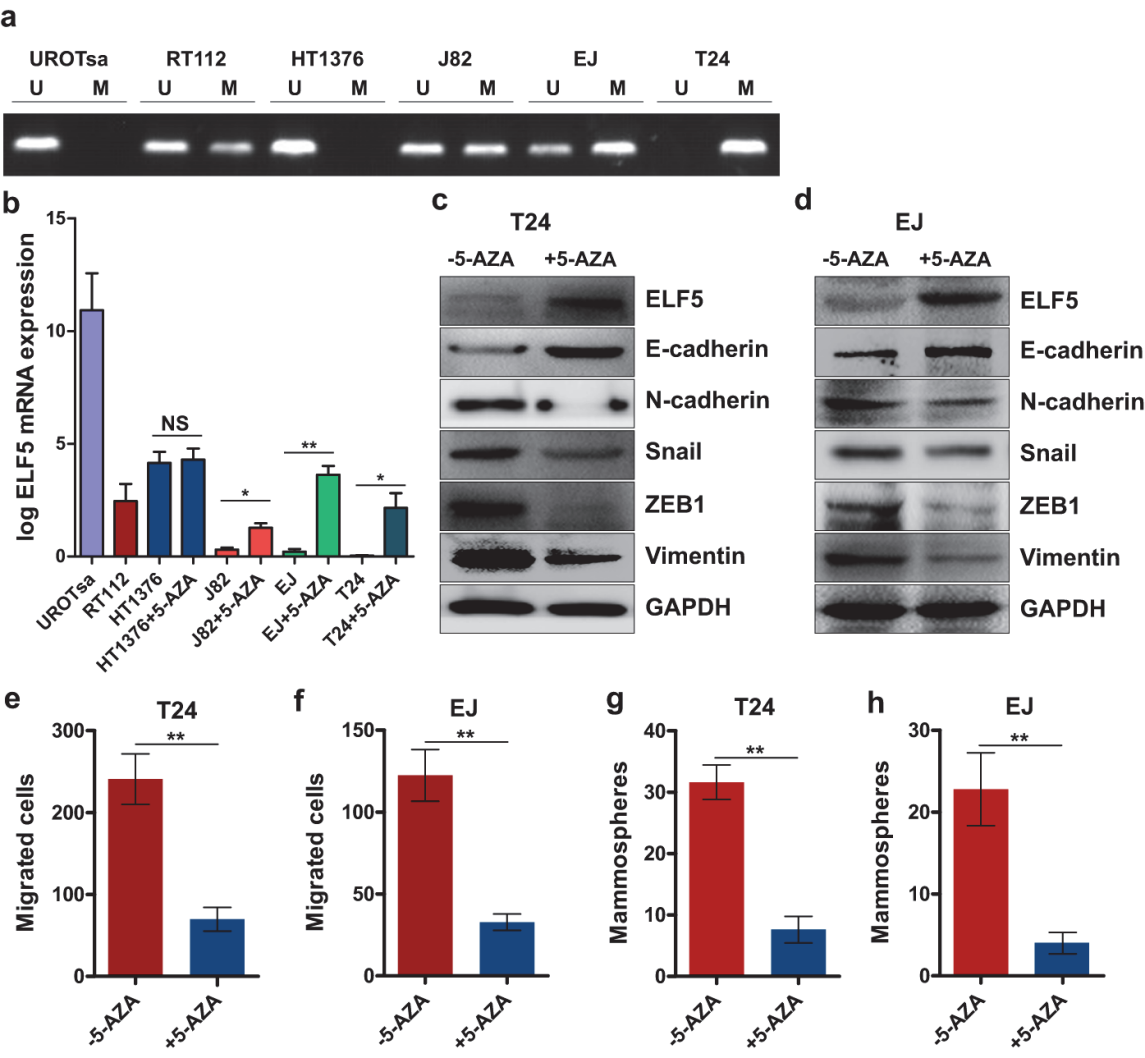
Elf5 promoter hypomethylation is associated with Elf5 expression and reverses mesenchymal characteristics of UC cells. a, Representative MSP results illustrating the Elf5 promoter methylation status of urothelial cell lines. b, Quantitative RT-PCR analysis the expression of Elf5 mRNA in parental UC cell lines and 5-AZA-treated HT1376, J82, EJ and T24. NS: not significant, *P < 0.05, **P < 0.01. c and d, Western blot analyses of Elf5 and EMT markers in EJ and T24 cells treated with or without 5-AZA. e and f, Migration assay: quantification of T24 as well as EJ treated with or without 5-AZA, n = 3. g and h, Sphere assay: quantification, n = 6. Data are presented as mean ±SEM, **P < 0.01.

Promoter methylation states in human bladder cancer cell lines were analyzed using MSP. The mesenchymal-like UC cell lines T24 and EJ revealed a hypermethylation in their promoter regions, whereas hypomethylation was detected in low-invasive HT1376 cells (Fig. 6a). We measured the level of Elf5 mRNA expression using RT-PCR assay. Doing so revealed higher Elf5 mRNA expressions in hypomethylated cell lines (Fig. 6b). To confirmed the relationship between promoter methylation status and Elf5 expression, we treated these UC cell lines HT1376, J82, EJ and T24 with 5μM demethylating agent 5-AZA. Elf5 mRNA expression was significantly restored after 5-AZA treatment except it was in hypomethylated HT1376 (Fig. 6b). In addition, Elf5 protein expression was also reversed by demethylation in T24 and EJ cell lines (Fig. 6c and 6d).

To determine whether the newly expressed Elf5 protein is functional, EMT makers and EMT-related transcription factors were analyzed by immunoblotting as well as invasion and sphere formation assays. We found epithelial cell marker increased and mesenchymal cell markers decreased after 5-AZA treatment in two mesenchymal-like UC cell lines (Fig. 6c and 6d). After 6 days of pretreatment, 5-AZA reduced 2.4-fold and 2.7-fold invasiveness in T24 and EJ cells, respectively (Fig. 6e and 6f). For sphere formation, 5-AZA respectively introduced 3.2-fold and 4.7-fold reduction in T24 and EJ cells (Fig. 6g and 6h). It is reasonable to conclude that promoter hypermethylation contributes to loss of Elf5 expression in UC cell lines.

## Discussion

Three significant observations were noted in this study. First, Elf5 is a prognostic factor in patients with UC. We showed that Elf5 expression was negatively correlated with tumor grade and stage. The early recurrence and disease-specific death were also observed in patients with low expression of Elf5. *In vitro*, low Elf5 expression is associated with high invasiveness, which can be effectively decreased by over-expressing Elf5 on purpose. Second, results from IHC staining of UC specimens revealed a negative relation between the transcription factor Elf5 and EMT, which is crucial for maintaining cellular traits associated with high-grade, invasiveness and recurrence. Moreover, this observation was demonstrated in cell lines by artificial regulation of Elf5 expression. Third, the Elf5 gene is hypermethylated in cancer cells, so that expansion of the mesenchymal compartment and subsequent SCs is induced and sustained.

Unlike mouse, the human Elf5 gene harbours four kinds of transcript variants with two alternative transcription start sites, one that identifies with orthologous start site of the mouse gene and gives rise to the Elf5–2b isoform, while the other one covered in the intron of Elf5–2b produces a longer variant Elf5–2a. Another transcript variant is spliced isoform of Elf5–2b with the ets consensus motif retained lacking of the coding exons 3 and 4 (SAM/Pointed domain), a widespread domain in signaling and nuclear proteins [13]. In recent study, the Elf5–2b variant was distinguished as a major form of Elf5 expressed in human, so this isoform was chosen in our study as well as in another paper, in which Elf5 was demonstrated to be an inhibitor of EMT in breast cancer [12].

Several studies have demonstrated that epigenetic regulation of Elf5 gene is a lineage gatekeeper between the embryonic and trophoblast lineage compartments, as well as between basal and luminal epithelial cells [14,26]. Here, we show that DNA methylation functions as a barrier of EMT in UC cells. The Elf5 promoter is CpG rich, but does not satisfy the criteria of a CpG island (http://cpgislands.usc.edu). Detailed characterization of the Elf5 promoter methylation profile by bisulphite sequencing analysis revealed that the vast majority of CpG residues in the 1-kb upstream region were methylated in cells with loss of Elf5 expression [14,26]. So, primers discerning sequence within this region were designed in this study. In additional data analysis, we found that the loss of Elf5 expression was not only restricted in patients with hypermethylation in Elf5 gene promoter. It is evident from the data that promoter methylation is not the sole determinant of Elf5 expression in UC cells. Previous studies have demonstrated hormone as an effective stimuli that can induce Elf5 expression in luminal epithelial cells, although mechanisms underlying these effects remain to be elucidated [27,28]. Additionally, some transcriptional repressors such as estrogen receptor (ER) may suppress Elf5 expression in the absence of promoter methylation, because we found that Elf5 is dominantly expressed in ER negative luminal epithelial cells [29], and a potential DNA binding site for ER has been identified near the Elf5 gene [30].

Clinically, the troublesome problem in regard to NMIBC is the unpredictability of recurrence and progression into muscle invasive disease, so it is necessary to look for efficient prognostic biomarkers for patients at high risk. In this study, Kaplan-Meier survival curves indicated that patients with positive expression of Elf5 less than 10% in UC had worse RFS after TURBT and DSS after radical cystectomy than those with more positive Elf5 expression. Increasing evidences suggest that anomalous EMT development triggers malignant tumor progression while Elf5 may have an inhibitory role in this process [12]. Reported evidence additionally revealed that Elf5-induced EMT inhibition is mediated by direct repression of the key inducer Snail [12]. Our study also represents proof that Elf5 is capable of down-regulating Snail in UC cell lines.

Together, our analyses conclude for the first time that the down-regulation of Elf5 by aberrant promoter methylation may promote bladder cancer recurrence in patients with primary NMIBC treated by TURBT. Moreover, our results suggest a supposed availability of Elf5 as a prognostic biomarker of disease-specific death for patients received radical cystectomy. In the physiological context, Elf5 is demonstrated to be functioned as a suppressor of EMT and cancer invasion. This may provide a new clue for novel target of UC therapy and valuable predictive biomarker to identify patients at high risk of recurrence and death from this disease.

## Supporting Information

S1 Fig (A and B) Kaplan-Meier curves of recurrence-free survival (RFS) according to Elf5 staining level in primary NMIBC treated by TURBT. (C and D), Kaplan-Meier curves of disease-specific survival (DSS) according to Elf5 staining level in UC patients after radical cystectomy.
(TIF)

S1 Table PCR primer sequences for Quantitative RT-PCR and MSP. (DOCX)

## References

[1] Acloque H, Adams MS, Fishwick K, Bronner-Fraser M, Nieto MA (2009) Epithelial-mesenchymal transitions: the importance of changing cell state in development and disease. J Clin Invest 119: 1438–1449. 10.1172/JCI38019 19487820 PMC2689100

[2] Thiery JP, Acloque H, Huang RY, Nieto MA (2009) Epithelial-mesenchymal transitions in development and disease. Cell 139: 871–890. 10.1016/j.cell.2009.11.007 19945376

[3] Yang J, Weinberg RA (2008) Epithelial-mesenchymal transition: at the crossroads of development and tumor metastasis. Dev Cell 14: 818–829. 10.1016/j.devcel.2008.05.009 18539112

[4] Singh A, Settleman J (2010) EMT, cancer stem cells and drug resistance: an emerging axis of evil in the war on cancer. Oncogene 29: 4741–4751. 10.1038/onc.2010.215 20531305 PMC3176718

[5] Zhou ZJ, Dai Z, Zhou SL, Hu ZQ, Chen Q, et al (2014) hnRNPAB induces epithelial-mesenchymal transition and promotes metastasis of hepatocellular carcinoma by transcriptionally activating Snail. Cancer Res. 10.1158/0008-5472.CAN-74-24-Reviewers 24638979

[6] Scheel C, Eaton EN, Li SH, Chaffer CL, Reinhardt F, et al (2011) Paracrine and autocrine signals induce and maintain mesenchymal and stem cell states in the breast. Cell 145: 926–940. 10.1016/j.cell.2011.04.029 21663795 PMC3930331

[7] Polyak K, Weinberg RA (2009) Transitions between epithelial and mesenchymal states: acquisition of malignant and stem cell traits. Nat Rev Cancer 9: 265–273. 10.1038/nrc2620 19262571

[8] Tam WL, Weinberg RA (2013) The epigenetics of epithelial-mesenchymal plasticity in cancer. Nat Med 19: 1438–1449. 10.1038/nm.3336 24202396 PMC4190672

[9] Taddei ML, Giannoni E, Comito G, Chiarugi P (2013) Microenvironment and tumor cell plasticity: an easy way out. Cancer Lett 341: 80–96. 10.1016/j.canlet.2013.01.042 23376253

[10] Wang Y, Shang Y (2013) Epigenetic control of epithelial-to-mesenchymal transition and cancer metastasis. Exp Cell Res 319: 160–169. 10.1016/j.yexcr.2012.07.019 22935683

[11] Kalyuga M, Gallego-Ortega D, Lee HJ, Roden DL, Cowley MJ, et al (2012) ELF5 suppresses estrogen sensitivity and underpins the acquisition of antiestrogen resistance in luminal breast cancer. PLoS Biol 10: e1001461 10.1371/journal.pbio.1001461 23300383 PMC3531499

[12] Chakrabarti R, Hwang J, Andres BM, Wei Y, Lukacisin M, et al (2012) Elf5 inhibits the epithelial-mesenchymal transition in mammary gland development and breast cancer metastasis by transcriptionally repressing Snail2. Nat Cell Biol 14: 1212–1222. 10.1038/ncb2607 23086238 PMC3500637

[13] Hemberger M, Udayashankar R, Tesar P, Moore H, Burton GJ (2010) ELF5-enforced transcriptional networks define an epigenetically regulated trophoblast stem cell compartment in the human placenta. Hum Mol Genet 19: 2456–2467. 10.1093/hmg/ddq128 20354077

[14] Ng RK, Dean W, Dawson C, Lucifero D, Madeja Z, et al (2008) Epigenetic restriction of embryonic cell lineage fate by methylation of Elf5. Nat Cell Biol 10: 1280–1290. 10.1038/ncb1786 18836439 PMC2635539

[15] Siegel R, Ma J, Zou Z, Jemal A. (2014) Cancer statistics, 2014. CA Cancer J Clin 64:9–29. 10.3322/caac.21208 24399786

[16] Rubben H, Lutzeyer W, Fischer N, Deutz F, Lagrange W, et al (1988) Natural history and treatment of low and high risk superficial bladder tumors. J Urol 139: 283–285. 3339726 10.1016/s0022-5347(17)42387-1

[17] Villicana P, Whiting B, Goodison S, Rosser CJ (2009) Urine-based assays for the detection of bladder cancer. Biomark Med 3: 265 20161673 10.2217/bmm.09.23 PMC2819730

[18] Donat SM (2003) Evaluation and follow-up strategies for superficial bladder cancer. Urol Clin North Am 30: 765–776. 14680313 10.1016/s0094-0143(03)00060-0

[19] Wu XR (2005) Urothelial tumorigenesis: a tale of divergent pathways. Nat Rev Cancer 5: 713–725. 16110317 10.1038/nrc1697

[20] Li H, Wang J, Xiao W, Xia D, Lang B, et al (2014) Epigenetic inactivation of KLF4 is associated with urothelial cancer progression and early recurrence. J Urol 191: 493–501. 10.1016/j.juro.2013.08.087 24018236

[21] Tian J, Lee SO, Liang L, Luo J, Huang CK, et al (2012) Targeting the unique methylation pattern of androgen receptor (AR) promoter in prostate stem/progenitor cells with 5-aza-2′-deoxycytidine (5-AZA) leads to suppressed prostate tumorigenesis. J Biol Chem 287: 39954–39966. 10.1074/jbc.M112.395574 23012352 PMC3501037

[22] Veeck J, Chorovicer M, Naami A, Breuer E, Zafrakas M, et al (2008) The extracellular matrix protein ITIH5 is a novel prognostic marker in invasive node-negative breast cancer and its aberrant expression is caused by promoter hypermethylation. Oncogene 27: 865–876. 17653090 10.1038/sj.onc.1210669

[23] Chakrabarti R, Wei Y, Romano RA, DeCoste C, Kang Y, et al (2012) Elf5 regulates mammary gland stem/progenitor cell fate by influencing notch signaling. Stem Cells 30: 1496–1508. 10.1002/stem.1112 22523003 PMC5606133

[24] Scheel C, Eaton EN, Li SH, Chaffer CL, Reinhardt F, et al (2011) Paracrine and autocrine signals induce and maintain mesenchymal and stem cell states in the breast. Cell 145: 926–940. 10.1016/j.cell.2011.04.029 21663795 PMC3930331

[25] Li LC, Dahiya R (2002) MethPrimer: designing primers for methylation PCRs. Bioinformatics 18: 1427–1431. 12424112 10.1093/bioinformatics/18.11.1427

[26] Lee HJ, Hinshelwood RA, Bouras T, Gallego-Ortega D, Valdes-Mora F, et al (2011) Lineage specific methylation of the Elf5 promoter in mammary epithelial cells. Stem Cells 29: 1611–1619. 10.1002/stem.706 21823211

[27] Hilton HN, Kalyuga M, Cowley MJ, Alles MC, Lee HJ, et al (2010) The antiproliferative effects of progestins in T47D breast cancer cells are tempered by progestin induction of the ETS transcription factor Elf5. Mol Endocrinol 24: 1380–1392. 10.1210/me.2009-0516 20519331 PMC5417466

[28] Menzies KK, Lee HJ, Lefevre C, Ormandy CJ, Macmillan KL, et al (2010) Insulin, a key regulator of hormone responsive milk protein synthesis during lactogenesis in murine mammary explants. Funct Integr Genomics 10: 87–95. 10.1007/s10142-009-0140-0 19830464

[29] Kendrick H, Regan JL, Magnay FA, Grigoriadis A, Mitsopoulos C, et al (2008) Transcriptome analysis of mammary epithelial subpopulations identifies novel determinants of lineage commitment and cell fate. BMC Genomics 9: 591 10.1186/1471-2164-9-591 19063729 PMC2629782

[30] Escamilla-Hernandez R, Chakrabarti R, Romano RA, Smalley K, Zhu Q, et al (2010) Genome-wide search identifies Ccnd2 as a direct transcriptional target of Elf5 in mouse mammary gland. BMC Mol Biol 11: 68 10.1186/1471-2199-11-68 20831799 PMC2949602

