# Supplementary figures and images for: Epigenetic Regulation of Elf5 Is Associated with Epithelial-Mesenchymal Transition in Urothelial Cancer
Source: PLoS One. 2015 Jan 28;10(1):e0117510. doi: 10.1371/journal.pone.0117510 (PMC4309403; doi:10.1371/journal.pone.0117510)

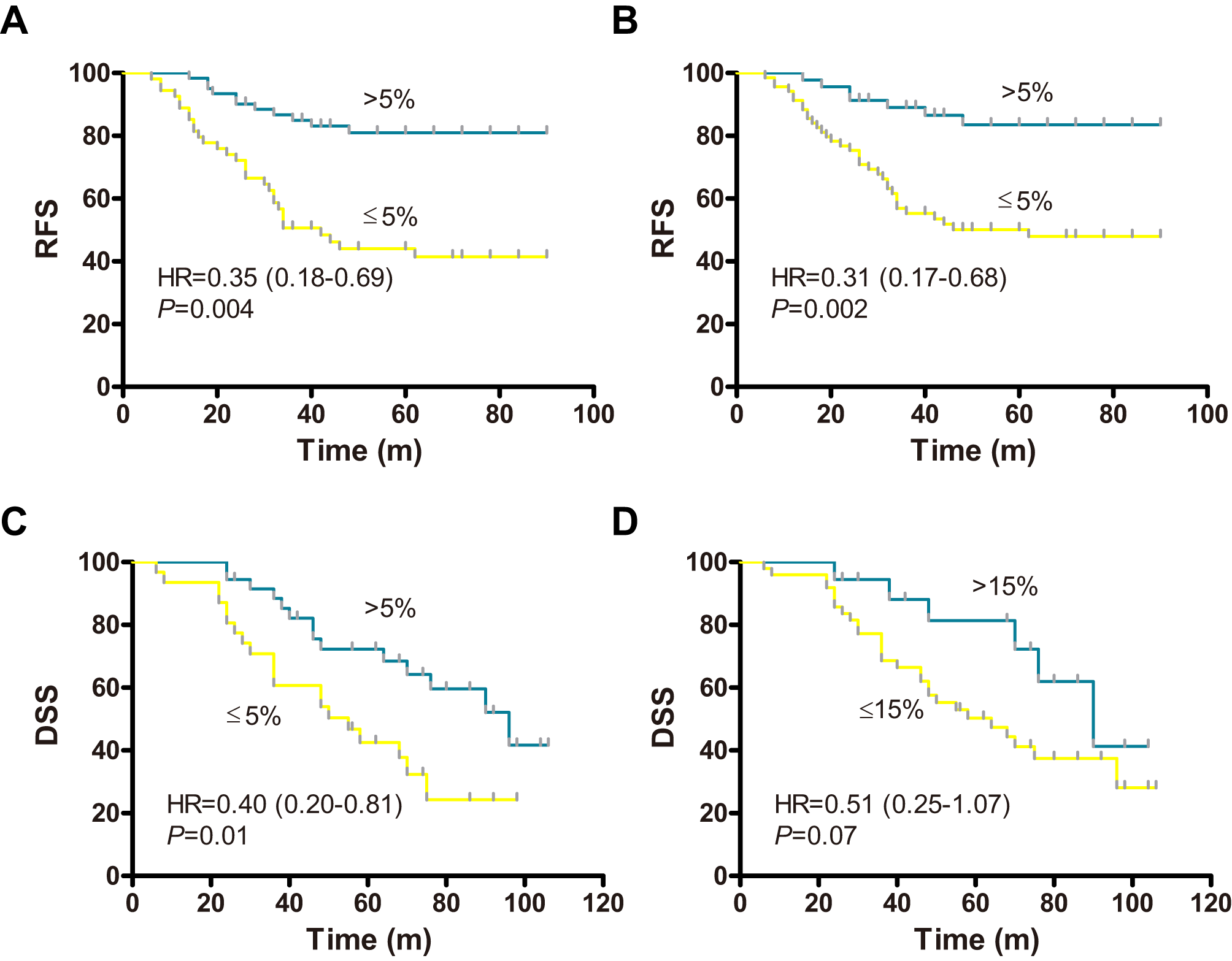

Supplement: S1 Fig — (C and D), Kaplan-Meier curves of disease-specific survival (DSS) according to Elf5 staining level in UC patients after radical cystectomy. (TIF) [file pone.0117510.s001.tif]
