# Supplementary material for: Epigenetic Regulation of Elf5 Is Associated with Epithelial-Mesenchymal Transition in Urothelial Cancer
Source: PLoS One. 2015 Jan 28;10(1):e0117510. doi: 10.1371/journal.pone.0117510 (PMC4309403; doi:10.1371/journal.pone.0117510)
Supplement: S1 Table — (DOCX) [file pone.0117510.s002.docx]

| **S1_table. PCR primer sequences for Quantitative RT-PCR and MSP** | | |
| --- | --- | --- |
|  |  |  |
| Gene | Forward | Reverse |
| *ELF5* | CGTGGACTGATCTGTTCAGCAATGA | CAGGGTGGACTGATGTCCAGTATGA |
| *GAPDH* | GGAGTCAACGGATTTGGTCGTA | GGCAACAATATCCACTTTACCAGAGT |
| *Methylated ELF5* | TAAAAATGTATTTGTAGGTTATGTGCG | ATTCTTACTTATTACCCAAACCGTC |
| *Unmethylated ELF5* | TAAAAATGTATTTGTAGGTTATGTGTGT | ATTCTTACTTATTACCCAAACCATC |
